# West Nile virus transmission in the Metropolitan Area of Barcelona (Spain): A One-Health surveillance approach

**DOI:** 10.1016/j.onehlt.2025.101150

**Published:** 2025-07-21

**Authors:** Núria Busquets, Jaume Gardela, Eduard José-Cunilleras, Alba Solé, Maria José Salvador, Elena Obón, Rafael Molina-López, Carles Aranda, Tomás Montalvo, Irene Corbella, Maria Assumpció Bou-Monclús, Miguel Julián Martínez, Ana Vázquez, Maria Piron, Sílvia Sauleda, Lola Pailler-García, Sebastián Napp

**Affiliations:** a IRTA, Programa de Sanitat Animal, Centre de Recerca en Sanitat Animal (CReSA), Campus de la Universitat Autònoma de Barcelona (UAB), Bellaterra, Catalonia, Spain; b Unitat mixta d'Investigació IRTA-UAB en Sanitat Animal, Centre de Recerca en Sanitat Animal (CReSA), Campus de la Universitat Autònoma de Barcelona (UAB), Bellaterra, Catalonia, Spain; c Servei de Medicina Interna Equina, Unitat Equina, Fundació Hospital Clínic Veterinari, Barcelona, Spain; d Departament d'Agricultura, Ramaderia, Pesca i Alimentació Generalitat de Catalunya, Servei de Prevenció en Salut Animal, 08007 Barcelona, Spain; e Centre de Fauna de Torreferrussa, Àrea de Gestió Ambiental Servei de Fauna i Flora, Forestal Catalana, Santa Perpètua de Mogoda, 08130, Spain; f Servei de Control de Mosquits del Consell Comarcal del Baix Llobregat, El Prat del Llobregat, Catalonia, Spain; g Servei de Vigilància i Control de Plagues Urbanes, Agencia de Salud Pública de Barcelona, 08023 Barcelona, Spain; h Centro de Investigación Biomédica en Red de Epidemiologia y Salud Pública (CIBERESP), Instituto de Salud Carlos III, 28029 Madrid, Spain; i Secretaria de Salut Pública, Departament de Salut, Generalitat de Catalunya, Barcelona, Spain; j Department of Clinical Microbiology, Hospital Clínic, Barcelona, Spain; k Centro de Investigación Biomédica en Red de Enfermedades Infecciosas (CIBERINFEC), Instituto de Salud Carlos III, Madrid, Spain; l Arboviruses and Imported Viral Diseases Laboratory, Centro Nacional de Microbiología, Instituto de Salud Carlos III, Madrid, Spain; m Transfusion Safety Laboratory, Blood and Tissue Bank of Catalonia, Servei Català de la Salut, Barcelona, Spain; n Centro de Investigación Biomédica en Red de Enfermedades Hepáticas y Digestivas (CIBEREhd), Instituto de Salud Carlos III, Madrid, Spain; o Transfusional Medicine Group, Vall d'Hebron Institut de Recerca (VHIR), Barcelona, Spain; p Departament de Medicina i Cirurgia Animals, Facultat de Veterinària, Universitat Autònoma de Barcelona, Bellaterra, 08193 Barcelona, Catalonia, Spain; q Barcelona Institute for Global Health (ISGlobal), Hospital Clinic de Barcelona, Universitat de Barcelona, Barcelona, Spain

**Keywords:** Arbovirus, Zoonosis, Vector-borne diseases, Public health surveillance, Mosquito vectors, Human health, Animal health, Flavivirus

## Abstract

West Nile virus (WNV), mainly transmitted by *Culex* mosquitoes, poses significant health risks to humans and horses, particularly in endemic regions. The first detection of WNV lineage 2 in Spain was in 2017 in Catalonia (northeastern Spain). In 2023, WNV was confirmed in a young yellow-legged gull and a probable human case was notified within the urban settings. We aimed to define the zone of WNV circulation in the Barcelona Metropolitan Area where these infections occurred and the effectiveness of the One Health approach for early WNV detection. The Catalan WNV surveillance and control programs includes the testing of horses, birds, mosquitoes and humans following molecular and serological methods. Phylogenetic analyses were performed to determine the origin of the circulating virus. IgM-positive data from both active and passive surveillance in horses identified the area of WNV circulation and suggested that WNV circulation happened either before or concurrently with human and bird infections in the agricultural and peri-urban areas. Furthermore, a new WNV introduction was discarded by phylogenetic studies, demonstrating that WNV lineage 2 has been established in Catalonia, albeit at a low level of circulation since the virus was not detected in blood donors. Our findings underscore the importance of integrating active and passive surveillance strategies to early assess WNV circulation and activate public health responses. The study highlights the role of wildlife in the WNV transmission and emphasizes the need for ongoing monitoring in animals and also mosquito control measures to mitigate the risk of animal and human infections.

## Highlights

• Horse IgM antibodies are useful to determine West Nile virus (WNV) circulation area.
• WNV monitoring in urban and non-urban animals are crucial to confirm WNV transmission.
• WNV lineage 2 had been established in Catalonia, albeit at low level of circulation.

## 1 Introduction

West Nile virus (WNV) (*Orthoflavivirus nilense*), a member of the *Orthoflavivirus* genus within the *Flaviviridae* family, is predominantly transmitted through the bite of infected *Culex* mosquitoes among birds as amplifying hosts [1]. Mammals, such as horses and humans, can be infected but are considered dead-end hosts. WNV in mammals is mainly diagnosed by serological methods, whereas WNV diagnosis in mosquitoes and birds is based on molecular detection [2,3]. Most WNV infections in horses are subclinical or mild, but neurological signs can appear such as ataxia or muscle fasciculations [4]. Humans can develop neurological signs leading to encephalitis, meningitis, or even death [5].

WNV has circulated in Europe for decades, with WNV lineage 1 being responsible for all outbreaks before WNV lineage 2 (WNV-L2) was first detected outside Africa in 2004, in a northern goshawk (*Astur gentilis*) in Hungary [6]. Since then, WNV-L2 has expanded across Europe [7,8]. In 2010, WNV cases were recorded in horses (36 infected herds) and humans (two cases) in the southwestern region of Spain (i.e., Andalusia) [9] caused by lineage 1, which has since become endemic [10]. A major outbreak with 77 human cases and 8 fatalities occurred in 2020, primarily in the southern Spanish province of Seville [11]. In 2024, an even more serious outbreak occurred. In this outbreak, the number of human cases was 138 [12] with 15 deaths [13] together with 68 cases in equids [12]. All WNV strains that were circulating in central and southwestern regions Spain up until 2024 has been determined to be WNV lineage 1 [14]. In 2017, WNV-L2 was first detected for the first time in the northeastern region of Spain (Catalonia) in a northern goshawk with neurological signs [15], and it has persisted in this region [16,17]. All these findings have been possible due to the WNV surveillance program in Catalonia that was initiated in 2007 [18].

The first confirmed human case was detected in Catalonia in 2001 [19], and it was not detected in humans again until 2022 [20], when an elderly couple displayed clinical signs of viral meningoencephalitis [21]. Between 2017 and 2022, WNV circulation was predominantly reported in animals (birds and horses) within agricultural and peri-urban areas across Catalonia [16]. However, in 2023, the epidemiological scenario changed with WNV circulation being identified in a yellow-legged gull bird and a suspected human case within the urban area (i.e., the Barcelona Metropolitan Area (BMA), which includes the city of Barcelona and surrounding municipalities with an area of 636 km^2^ and a population of 3.2 million people [22]. Following confirmation of the WNV circulation, the Regional Health authorities deployed the public and animal health measures as described in the protocols for WNV surveillance and control, increasing the awareness among veterinarians and clinicians [23]. In the study, in order to establish the area of WNV circulation, an active surveillance was performed in horses, in blood donors and mosquitoes in the BMA. Additionally, we aimed to determine the origin of the virus and estimate the time of WNV circulation by phylogenetic analysis of WNV isolates from the WNV infected yellow-legged gull and by tracing IgM antibodies in horses, respectively. Overall, we assessed the effectiveness of several One Health surveillance activities for early WNV detection.

## 2 Methods

### 2.1 Surveillance of WNV in the BMA

In 2023, the Catalan surveillance and control programs, which are coordinated by the Department of Health and the Department of Agriculture, Livestock, Fisheries and Food, involved the surveillance of birds, horses, humans and mosquitoes [24,25]. This surveillance was mainly performed during the mosquito vector activity (from mid-June to mid-November). Ethical approval for sampling was not necessary since the data was obtained from routine surveillance for public health authorities. The procedures were carried out in accordance with approved guidelines, regulations and the principles of the Declaration of Helsinki.

Passive surveillance (i.e., animals with clinical signs compatible with WNV or found dead) in wild birds was performed mainly in collaboration with Wildlife Recovery Centres (WRC). Dead bird heads from Barcelona province were collected at the WRC and transported to IRTA-CReSA for molecular WNV testing under biosafety level 3 (BSL3) conditions.

After WNV detection in a bird (yellow-legged gulls (*Larus michahellis*)) and a human WNV case was suspected, WNV surveillance in horses and in mosquitoes was established near areas of detected WNV circulation.

The Equine Unit of the Veterinary Clinic Hospital together with equine veterinarians collected passive and active surveillance samples in horses. Active WNV survey in horses covered two of the six counties of the BMA. The first includes the city of Barcelona on the Mediterranean coast and four surrounding municipalities, covering an area of 146 km^2^ that is predominantly urban and with a population of 2.3 million [22]. The second county, Baix Llobregat, comprises 30 municipalities with a population of 486,000 over an area of 486 km^2^, featuring a mix of urban, wetlands, forested and, agricultural areas [22]. Blood samples from symptomatic horses and active surveillance were also tested at IRTA-CReSA using serological techniques.

**Fig. 1:**
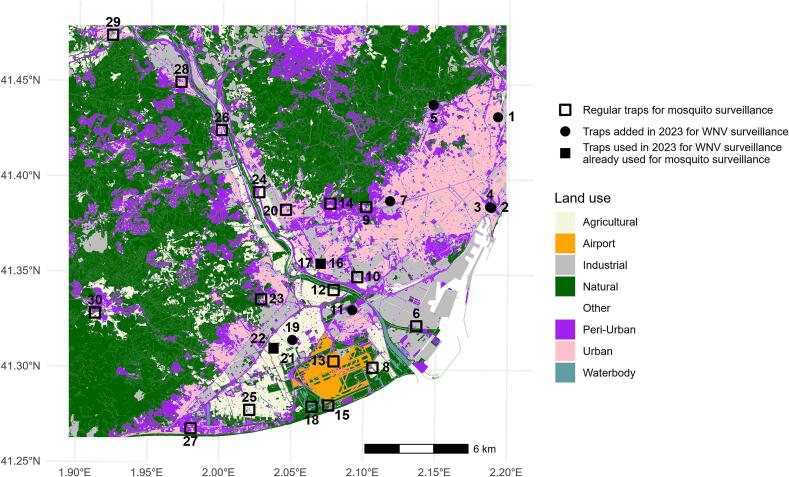
Location of the regular mosquito traps for mosquito surveillance (empty squares), mosquito traps added in 2023 for WNV surveillance (black circles), and mosquito traps previously used for mosquito surveillance that were used in 2023 for WNV detection (black squares).

Mosquitoes were collected using four Encephalitis Virus Surveillance (EVS)-CO_2_ traps placed near areas of detected WNV circulation, two of these traps already installed in the Baix Llobregat county. Moreover, based on mobility data of yellow-legged gulls [26], six BG (Biogents) traps were placed in Barcelona (Fig. 1). Mosquito detection was focused on females of the *Cx. pipiens* species, the main vector of WNV in the region. Mosquitoes were collected in one night, taxonomically classified, and *Cx. pipiens* were pooled in groups of up to 25 females for molecular analysis. To assess whether *Cx. pipiens* abundance influenced WNV circulation in the BMA area in 2023, data from twenty EVS-CO_2_ traps located in different environments by the Mosquito Control Service (MCS) in the Baix Llobregat, especially in the Llobregat Delta (Fig. 1), were analysed. Mosquitoes were collected one night every two weeks from week 18 to week 46. The female *Cx. pipiens* abundance was compared between two periods (pre and post WNV detection), from week 18 to 28, and from week 30 to 46, respectively, along environments. All statistical analyses were performed with R Studio and using the Wilcoxon test with a Bonferroni correction for multiple comparisons.

Human passive surveillance mainly consisted in screening for WNV in individuals with compatible neurological disorders. Hospitals in the affected area were instructed to intensify passive case finding. Besides, all blood and tissue donors in Catalonia were individually screened for WNV.

### 2.2 WNV detection by molecular methods

Mosquito pools and brains from dead birds were homogenized by using a bead mill (TissueLyser II, Qiagen, Denmark) at 30 Hz for 1 min and polypropylene pestles, respectively. Viral RNA was extracted using NucleoSpin® RNA Virus (Macherey-Nagel, Düren, Germany) according to the manufacturer's instructions. WNV RNA was detected by real-time reverse transcription PCR (RT-qPCR) using the primers and probe previously described [27] and AgPath-ID™ OneStep RT-PCR reagents (Applied Biosystems, Foster City, CA, USA). The amplification was performed using a Real-Time PCR 7500 Fast System (Applied Biosystems, Foster City, CA, USA).

Blood and tissue donations from the Blood and Tissue Bank of Catalonia were routinely tested on the Procleix Panther System for WNV RNA using the Procleix WNV assay (Grifols, San Diego, CA).

Further analyses to determine the WNV lineage were performed in positive samples by using the primers and probes described by Jimenez-Clavero and collaborators [28] and AgPath-ID™ OneStep RT-PCR reagents (Applied Biosystems, Foster City, CA, USA). The amplification was detected using a Real-Time PCR 7500 Fast System (Applied Biosystems, Foster City, CA, USA).

### 2.3 Detection of flavivirus and West Nile virus circulation by serological methods

Serological blood tests for humans, using WNV IgM Capture DxSelect and WNV IgG DxSelect ELISA kits (Focus Diagnostics, Cypress, California, USA), were carried out at the Clinical Microbiology Department of Hospital Clinic of Barcelona and at the Spanish National Centre of Microbiology. To confirm the specificity of the antibody response, positive or indeterminate sera in both WNV IgG and IgM ELISA tests were assayed by virus neutralization test (VNT) against WN (strain HU6365/08) and Usutu virus (USUV) (strain HU10279/09) viruses [29].

Regarding serological blood testing in horses, about 2 mL of equine blood was collected in non- anticoagulant tubes. The blood samples were centrifuged at 2500 rpm during 10 min to obtain serum samples and were kept at −20 °C until serological testing. Equine serum samples were tested using the commercially available competitive enzyme linked immunosorbent assay (cELISA) for the specific detection of antibodies to flaviviruses (INGEZIM West Nile COMPAC kit #10.WNV·K3, Ingenasa, Spain). Positive sera were re-tested using a capture ELISA for detection of WNV IgM specific antibodies in equine serums (INGEIM West Nile IgM kit #14.WNV·K2, Ingenasa, Spain). Positive samples for cELISA were sent to the Central Veterinary Laboratory in Algete (Madrid) for confirmation by VNT against WNV (Morocco 03 strain). In IgM-positive horse cases, microtitre VNT was also performed for Usutu virus (SAAR 1776 strain). Neutralization test is described in the WOAH Terrestrial Manual [2].

### 2.4 Detection and duration of IgM antibodies in horses for monitoring WNV spread in the BMA

A WNV survey in horses was designed to detect recent infections by IgM detection [2]. Horse farms within 10 km of bird and human WNV cases were selected, with sample sizes calculated to detect at least 20 % within-herd prevalence at 95 % confidence level, testing up to 13 horses per herd. Inclusion criteria for horses included those not vaccinated against WNV and those that had not travelled to WNV-endemic areas during at least the last two months prior to sampling as well as herds near stagnant water. If any horse tested IgM-ELISA positive for WNV, additional equine farms around were tested until all surrounding farms resulted negative.

To better define the timing of recent WNV circulation and IgM duration, IgM-positive horses during 2023 were followed up and tested again after 2 to 4 weeks when possible.

### 2.5 Virus isolation

WNV isolation from positive bird was performed in Vero cells (ATCC, ref. CCL-81) at the IRTA-CReSA BSL3 facilities. Cells at 90 % confluence were exposed to homogenized sample supernatant for 1 h and then incubated for 4 days in post-infection media (Dulbecco's Modified Eagle Medium with 2 % foetal bovine serum, 2 % antibiotic-antifungal (penicillin (10.000 units/mL), streptomycin (10,000 μg/mL) and amphotericin B (25 μg/mL), and 2 % glutamine, all from GIBCO). The supernatant was then collected, centrifuged, aliquoted, and stored at −80 °C. WNV isolation was confirmed by observing cytopathic effect and decreasing Ct values, from Ct of 25.76 in brain sample to a Ct of 20.88 in one aliquot of the supernatant using RT-qPCR as described above for WNV detection using primers and probe designed previously [27].

### 2.6 Full genome sequencing and phylogenetic analysis

Total RNA was extracted from 200-μL aliquots of infected cell culture supernatants (1st passage) using TRi-reagent (Sigma-Aldrich) and following the manufacturer's instructions.

RNA samples were checked using a Nanodrop (Thermo Fisher Scientific) and quantified with the Qubit ssRNA (Thermo Fisher Scientific). Library preparation was performed following the NEBNext Ultra II Directional RNA Library Prep Kit for Illumina (NEB #E7760S, New England Biolabs) starting with a total of 100 ng without any enrichment step. The final libraries were checked using a Bioanalyzer dsDNA High Sensitivity (Agilent). Sequencing was performed on a MiSeq instrument (Illumina) using MiSeq Reagent Nano Kit v2, paired-end (2 × 150 bp) and 300 cycles (Q30 (90,61 %) and 90,19 % Passing Filter). The obtained reads were processed using the SPAdes Genome Assembler software (Algorithmic Biology Lab, version 3.6.0) from the Galaxy platform (usegalaxy.org, version 3.15.5, *rnaviralSPAdes* tool). GenBank access number: PQ641624.

For phylogenetic analysis, complete WNV sequences were aligned with homologous sequences from other representatives WNV-L2 strains obtained from GeneBank (https://www.ncbi.nlm.nih.gov/genbank/, accessed on 26 July 2024). Alignments were performed using MAFFT version 7. The aligned sequences were then inspected and curated using MEGA version 11 [30]. Phylogenetic trees were constructed using the Maximum Likelihood Method in MEGA version 11 [30]. Bootstrap analyses were inferred from 1000 replicates.

## 3 Results

### 3.1 WNV outbreak in the BMA

**Fig. 2:**
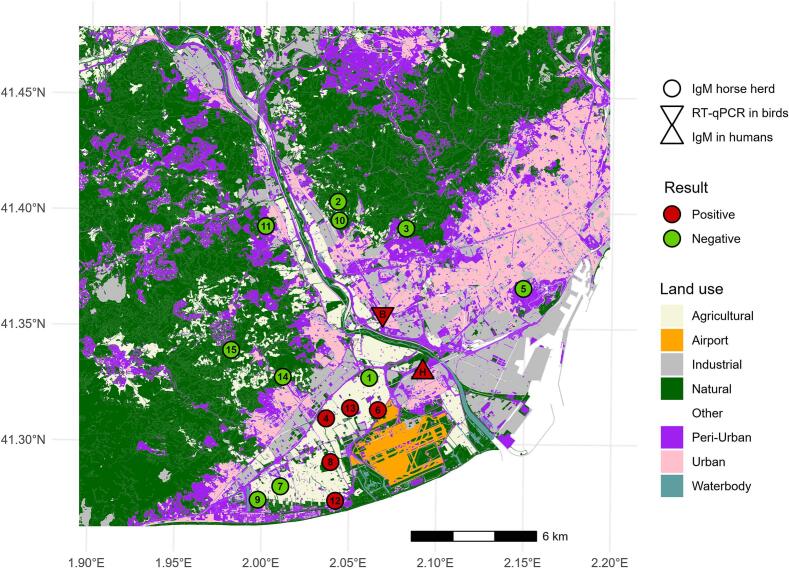
A) Distribution of the West Nile virus (WNV) outbreak in the Barcelona Metropolitan Area in 2023. The red triangle marks the location of the WNV-positive yellow-legged gull, the inverted red triangle indicates the probable human case, and the circles show the WNV IgM-positive (red) or WNV IgM-negative (green) horse herds. B) Timeline of the WNV outbreak. The top section illustrates suspected cases (black arrows) and confirmed or probable cases (red arrow) detected by passive surveillance in birds, horses and humans. The middle section represents the equine serosurvey, with red squares denoting WNV IgM-positive horse herds and green squares representing WNV IgM-negative herds. The bottom section shows mosquito surveillance, with all samples testing WNV RT-qPCR negative (green). The number within the squares correspond to the order of sampling sequence shown in Fig. 2A. (For interpretation of the references to colour in this figure legend, the reader is referred to the web version of this article.)

In 2023, 47 sick or dead wild birds were collected in the BMA. On July 21, a young yellow-legged gull was found weak in an urban area of Cornellà de Llobregat (Fig. 2). The gull exhibited severe depression, dropped head, and ataxia. It was euthanized and the head was sent to the laboratory on July 24. On July 28, the gull was confirmed as WNV-positive and identified as WNV-L2 by specific RT-qPCR. The remaining 46 birds tested negative.

On August 18, the epidemiologists of the BMA received the notification of a probable WNV human case, residing in the urban area of El Prat de Llobregat, approximately 3.3 km from the location where the positive gull was found (Fig. 2). The patient was a 77-year-old woman receiving immunosuppressive therapy following a kidney transplant six years earlier. Her initial symptoms - weakness and gastrointestinal distress including, abdominal pain, vomiting, and diarrhoea- began on July 27. Eight days later, she developed persistent fever and encephalitic symptoms characterized by bradypsychia and somnolence. Neuroimaging and electroencephalography revealed findings consistent with encephalitis. Cerebrospinal fluid (CSF) analysis demonstrated lymphocytic pleocytosis with a leucocyte count of 117 cells/mm^3^ (neutrophils 6.9 %, lymphocytes 77 %, monocytes 1.2 %), glucose concentration of 37 mg/dL, adenosine deaminase activity of 7 U/L and protein level of 70 mg/dL. Other pathogens -including *Mycobacterium tuberculosis*, *Neisseria meningitidis*, *Streptococcus pneumoniae*, *Streptococcus agalactiae*, *Listeria monocytogenes*, and *Cryptococcus neoformans*- as well as neoplastic processes were excluded. Testing for viral infections such as cytomegalovirus, respiratory viruses and especially other arboviruses (Toscana virus and USUV) was negative. The CSF volume was insufficient for further virologic analysis; thus, diagnosis relied on repeated serologic testing of serum samples.

WNV-specific IgM antibodies were consistently detected in serum during both the acute and the convalescence phases, while IgG antibodies were never detected, and the specific real-time PCR assay (RT-PCR) was negative in serum and urine. VNT were performed in serum samples at 25 and 69 days after symptoms onset, resulting in the first one being indeterminate (titer 1/8) and the second one negative for WNV. Both samples were negative for USUV NT assay, and the serum sample at day 25 tested negative for both IgM and IgG against Toscana virus, discarding other possible arbovirus infection. Therefore, the case was classified as a WNV probable case.

Following hospitalization, including intensive care, the patient underwent cognitive and functional rehabilitation at a socio-healthcare centre and at home for six months. She remains with residual sequelae, notably chronic polytopic pain, amnesic dysfunction, emotional disturbances, disability and dependence in activities of daily living.

Universal screening of WNV in individual samples were performed in 79,579 blood donations and in 369 cadaveric tissue donors, all tested negative. Also, no organ donor was excluded.

Following WNV detection in the gull, passive surveillance in horses was activated urging the association of equine veterinarians of Catalonia to communicate to the animal health authorities any horse with signs compatible with WNV infection. As a result, on September 5, a horse in Viladecans showed signs of ataxia (paralysis-paresis) and was confirmed IgM-positive on September 13 (Fig. 2).

Active WNV surveillance in mosquitoes began on August 9 and ended on October 30 with 10 mosquito traps (Fig. 1, Fig. 2). A total of nineteen captures were carried out in which 335 *Cx. pipiens* females from 37 pools tested negative for WNV.

### 3.2 Assessing the expansion of WNV in the BMA

Between August 3 and October 9, 14 horse farms (125 horses) surrounding the human, bird and horse cases were evaluated (Fig. 2). Of these, 13 farms were in Baix Llobregat, and one equestrian school was in Barcelona city. Of the 14 farms, four of them have IgM-positive horses, with a total of eight horses being positive. Of these eight horses, four showed specific antibodies against WNV by VNT (≥1/20) (Table 1), indicating a recent WNV infection. Three farms with IgM-positive horses were in Viladecans, and one was in El Prat de Llobregat, near the probable human case. All farms with IgM-positive horses were in agricultural (2/3, 40 %), natural (1/2, 50 %) and peri-urban (2/5, 20 %) areas (Fig. 2). The remaining ten farms, located to the west, north and east of the WNV-positive bird and the probable human case, tested IgM-negative. However, six out of the ten remaining farms were cELISA-positive, suggesting previous exposure to a flavivirus. Of these, four had VNT results against WNV ranging from 1/10 to 1/32, suggesting possible, but not recent, WNV exposure. The remaining two positive herds for cELISA but negative for VNT against WNV indicate an exposure to a flavivirus different to WNV.

**Table 1:** Virus neutralization test (VNT) results in IgM-positive horses.

Municipality	Sampling date	WNV VNT titer	USUV VNT titer	WNV result
Viladecans	3/8/2023	<1/5	N.A.	Negative
Prat de LLobregat	8/8/2023	1/20	<1/5	Positive
Prat de LLobregat	8/8/2023	1/5	1/5	Negative
Viladecans	21/8/2023	Cytotoxic effect	<1/5	No conclusive
Viladecans	7/9/2023	1/20	<1/5	Positive
Viladecans	7/9/2023	1/20	1/5	Positive
Viladecans	13/9/2023	1/20	<1/5	Positive
Viladecans	13/9/2023	1/10	<1/5	Negative

N.A. Not available.

### 3.3 Abundance of WNV vector (*Cx. pipiens*) in the BMA

**Fig. 3:**
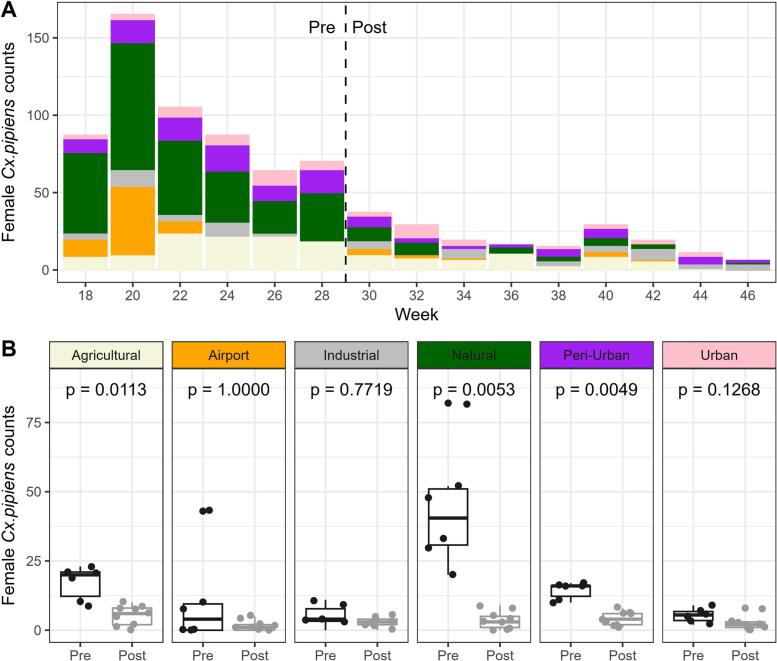
Counts of *Cx. pipiens* females collected with EVS-CO_2_ traps by the Mosquito Control Service in different environments from week 18 to week 46, from 2021 to 2023. Pre and post indicate the period before and after WNV detection in the yellow-legged gull. (For interpretation of the references to colour in this figure legend, the reader is referred to the web version of this article.)

Results on mosquito abundance indicated that the counts of female *Cx. pipiens* were generally low (<100 mosquitoes/capture) (Fig. 3A). However, before WNV detection in the young yellow-legged gull (week 29), the number of *Cx. pipiens* females was significantly higher in natural, peri-urban and agricultural areas in comparison with the number of *Cx. pipens* females after the detection (Fig. 3B), whereas no similar trend was observed in other areas such as urban area.

### 3.4 Duration of IgM antibodies against WNV

**Fig. 4:**
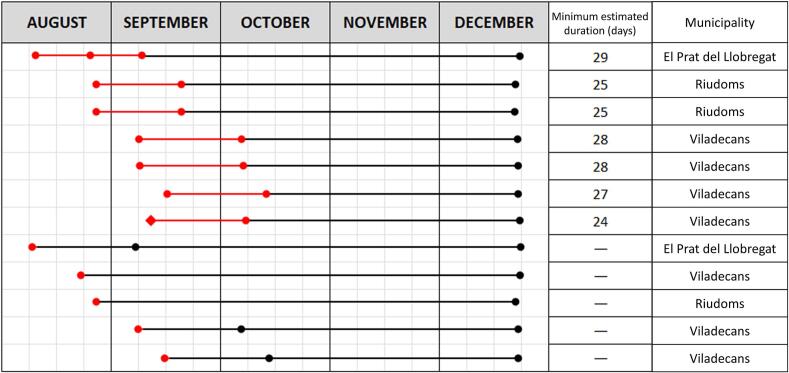
The dates of positive (red) and negative (black) WNV IgM results in individual horses from the study in the BMA. Red diamond represents sample obtained by passive surveillance. (For interpretation of the references to colour in this figure legend, the reader is referred to the web version of this article.)

Twelve IgM-positive horses were collected across several areas of Catalonia in 2023, and multiple samples were collected from 6 of them. This allowed for the estimation of IgM antibody duration, which ranged from 24 to 29 days (Fig. 4), with an average duration of 26.6 days (95 % CI: 24.8 to 28.3 days). In one additional case, the duration was estimated based on the onset of clinical signs, as IgM antibodies typically peak in the early days of clinical disease [4]. All samples tested negative by December, with three already negative in September and October.

### 3.5 Phylogenetic study

**Fig. 5:**
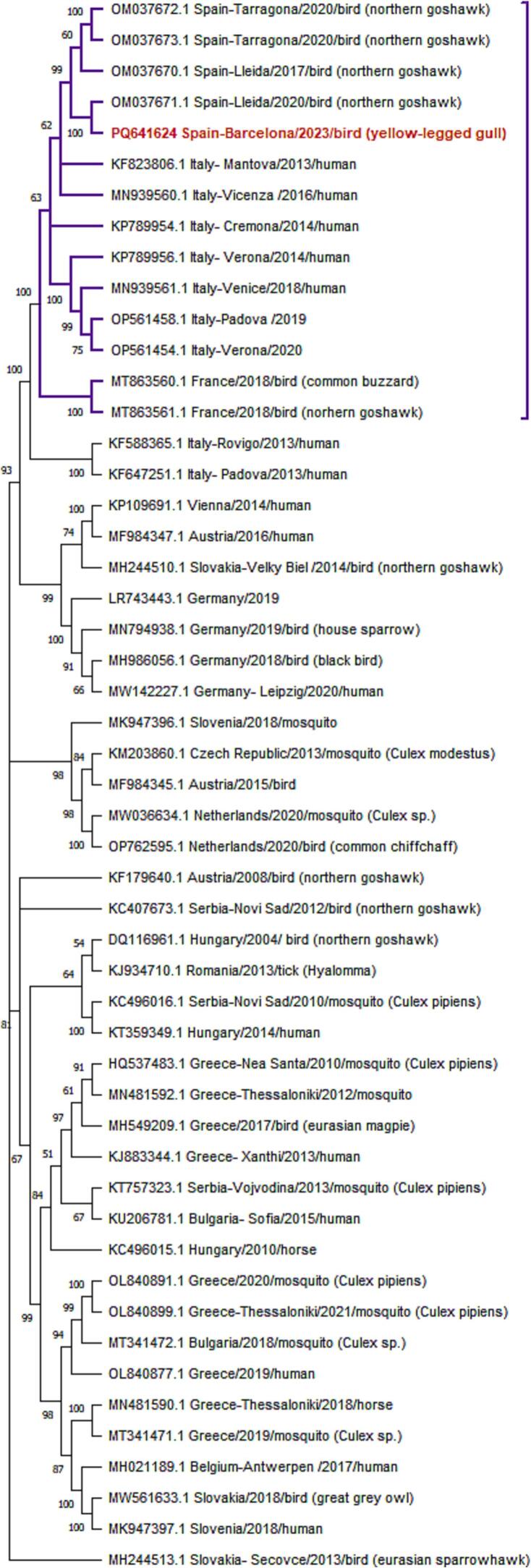
Phylogenetic tree of West Nile virus lineage 2 isolates constructed using the Maximum Likelihood method and Tamura-Nei model [31]. The tree with the highest log likelihood (−21,778.50) is shown, with bootstrap support values displayed next to branches. The Italian Lombardy cluster, highlighted in purple, includes our 2023 isolate (AC1720, which has the following GenBank code number: PQ641624 and is highlighted in red). The tree is scaled to substitutions per site using 51 nucleotide sequences and10312 positions. Evolutionary analyses were conducted in MEGA11 [30]. (For interpretation of the references to colour in this figure legend, the reader is referred to the web version of this article.)

A phylogenetic analysis compared fifty WNV-L2 isolates, including recent ones from main recognized clades in Europe, with the 2023 isolate (AC1720), which has the following GenBank code number: PQ641624. The WNV-L2 isolate AC1720 belonged to the central-southern European WNV lineage 2 clade (Hungarian clade) and was closely related to isolates from Italy (2013−2020) and France (2018), forming a monophyletic group known as the Italian Lombardy cluster (Fig. 5). Isolate AC1720 showed a closer genetic relationship to Catalan isolates, particularly the 2020's isolate from Lleida province.

## 4 Discussion

Since the first detection of WNV-L2 in Catalonia in 2017 [15], the virus has been able to persist and spread to new areas, mainly concentrating in agricultural and peri-urban areas [16]. In 2023, the epidemiological situation became more complex, as WNV was detected in a bird and one probable human case was notified in urban areas within the BMA. Although the results of our study based on active surveillance in horses indicate that WNV circulation was also in other environmental areas such as agricultural and peri-urban areas.

To determine the effectiveness of any measures to limit the risk posed by WNV, it is key to establish where and when WNV circulation occurs. Our investigation in the BMA suggests that the most probable scenario involves the amplification of WNV by competent bird species in some of the agricultural and peri-urban areas surrounding Barcelona and neighbouring municipalities, as WNV infections were detected in five IgM-positive horse herds and one symptomatic horse was confirmed in municipalities characterized by an abundance of wetlands and agricultural areas, particularly irrigated areas [32]. In these locations, the most common species are passerines, although the yellow-legged gull is also abundant [33] providing adequate conditions for WNV amplification. The relevance of the agricultural areas for WNV transmission coincides with the findings reported by Lu and collaborators [8], who pointed out that agricultural land use may be a significant driver in the emergence and spread of WNV. From those hot-spots, expansion into the actual urban areas seems possible, potentially via bridge birds.

The reported WNV-positive bird was a juvenile yellow-legged gull, a common bird species in the city of Barcelona [34,35]. Yellow-legged gulls are potential pathogen dispersers in Barcelona [26], with differences in behaviour between immatures and adult individuals. Immature birds are more likely to travel beyond Barcelona's boundaries and exploit a diverse range of trophic resources [26]. A 2018 study found that yellow-legged gull chicks in Barcelona feed on fishery discards and urban birds [36], including passerines, which are highly competent for WNV [37]. Management measures and changes in resource exploitation in the city have significantly reduced the intake of organic waste as food for gulls [36], potentially increasing their consumption of urban birds, some of which may be a source of WNV infection [38]. The Laridae family is highly susceptible to WNV [39], as evidenced by reports of yellow-legged gull mortality during the 2018 WNV outbreak in Israel [40]. Yellow-legged gulls are also the most abundant bird species in the Delta del Llobregat Natural Park [41], where they are in contact with both migratory and resident birds. Considering their competence of WNV, their abundance in urban and agricultural areas, their interaction with other bird species, and their tendency to disperse, especially for immature individuals, the yellow-legged gull is an ideal candidate for passive surveillance and an interesting species for WNV epidemiology studies.

The detection of a WNV-L2 strain in 2023 phylogenetically close to those previously detected in Catalonia confirms that the virus has been circulating since then, being detected this time about 150 km away from the area (Lleida province) where the phylogenetically closest WNV-L2 strain was detected in 2020. The dispersal velocity is slower than the estimated for WNV-L2 (88km/yr–215 km/yr) [8], which may indicate a silent circulation during the two previous years. This finding would discard a new introduction from migratory birds. Moreover, even though WNV circulation seemed to occur in the nearby agricultural areas, both the infected bird and the probable human case were found within the urban areas of the BMA, which suggests that autochthonous transmission within these areas is possible, as demonstrated in Berlin [42].

WNV surveillance based exclusively on data obtained passively surely underestimates the WNV extension. Our serosurvey results in horses delimited the area of recent WNV circulation for control strategies and assessing the duration of IgM allowed to estimate the onset of the infection. The mean minimum IgM duration of 26.6 days agrees with previous estimates that indicated durations of 1–2 months [2], so that we could infer that in these horses WNV transmission occurred between the June 6 and August 9, and is consistent with the WNV detection in both the gull and the probable human cases. These results confirm that IgM detection may be useful in determining the WNV circulation area, mainly in areas with a recent WNV introduction or unvaccinated horses that could be used as sentinels.

Several factors associated with mosquito-borne disease transmission, such as local abundance of competent vectors, have been proposed to explain the spatially non-random occurrence of disease outbreaks [43]. Our results show that *Cx. pipiens* counts in 2023 were quite low, but in natural, agricultural and peri-urban areas the number of mosquitoes were higher at the beginning of the season, before WNV detection compared to the later weeks. This may suggest that mosquitoes in these areas could have contributed to WNV transmission in wild birds present in those areas. This hypothesis aligns with the period preceding WNV infection in the yellow-legged gull, the WNV probable infection in the patient, and the estimated WNV infection in horses. The decreased mosquito numbers later in the season indicates that the prevention and control measures implemented in 2023 were effective in reducing the mosquito population.

The WNV surveillance and control programs in Catalonia under the One Health approach have demonstrated their effectiveness by detecting WNV in birds and horses before human cases occurred. This was evident in Reus (Tarragona), where WNV-positive birds and horses were detected in 2020, followed by WNV-positive birds, horses and mosquitoes in 2021 [20], and finally, in 2022, the first two human cases were detected [21]. WNV surveillance in mosquitoes has been effective in other regions such as Italy [44] or southern Spain [45]. However, we were unable to detect WNV in mosquitoes, likely due to the low level of viral circulation and the low numbers of *Cx. pipiens* females when the mosquitoes were collected.

WNV surveillance strategies that are effective in some epidemiological situations may not work as well in others. In north-eastern Spain, where WNV is caused by lineage 2, detection of cases has relied hugely on passive surveillance in birds, mainly in northern goshawk [17]. One key advantage of this type of detection is that the delay between suspicion and confirmation using RT-qPCR is very short, which is essential for control measures. That was also the case of the gull in BMA in 2023 that took only 7 days to be confirmed. Detection based on serological tests requires confirmation by VNT, which is more time-consuming and complex because of cross-reactions with other flaviviruses.

Within the framework of the One Health approach, passive surveillance of wild birds and IgM detection in unvaccinated horses or those not previously exposed to WNV should be prioritized as key components of WNV surveillance in the coming years in Catalonia and any other regions where WNV is not endemic or has emerged over the last years. Juvenile yellow-legged gulls should be the key target species, such as other susceptible species, like northern goshawks. Moreover, the implementation of the integrated mosquito population control by the public MCS is crucial to prevent or mitigate the WNV circulation in regions with WNV vectors during periods of mosquito activity.

## CRediT authorship contribution statement

**Núria Busquets:** Writing – original draft, Supervision, Resources, Project administration, Methodology, Investigation, Funding acquisition, Formal analysis, Data curation, Conceptualization. **Jaume Gardela:** Writing – review & editing, Investigation, Formal analysis. **Eduard José-Cunilleras:** Writing – review & editing, Methodology, Investigation. **Alba Solé:** Writing – review & editing, Resources, Methodology, Investigation. **Maria José Salvador:** Writing – review & editing, Resources, Methodology, Investigation. **Elena Obón:** Writing – review & editing, Methodology, Investigation. **Rafael Molina-López:** Writing – review & editing, Methodology, Investigation. **Carles Aranda:** Writing – review & editing, Methodology, Investigation. **Tomás Montalvo:** Writing – review & editing, Methodology, Investigation. **Irene Corbella:** Writing – review & editing, Resources, Methodology, Investigation. **Maria Assumpció Bou-Monclús:** Writing – review & editing, Resources, Methodology, Investigation. **Miguel Julián Martínez:** Writing – review & editing, Methodology, Investigation. **Ana Vázquez:** Writing – review & editing, Methodology, Investigation. **Maria Piron:** Writing – review & editing, Methodology, Investigation. **Sílvia Sauleda:** Resources, Investigation. **Lola Pailler-García:** Writing – review & editing, Visualization, Investigation, Formal analysis, Data curation. **Sebastián Napp:** Writing – original draft, Visualization, Project administration, Methodology, Investigation, Funding acquisition, Formal analysis, Data curation, Conceptualization.

## Funding details

This work was supported by the 10.13039/100014440Ministerio de Ciencia, Innovación y Universidades under Grant PID2020-116768RR-C22; the 10.13039/501100010552Departament de Salut, Generalitat de Catalunya; and the Departament d'Agricultura, Ramaderia, Pesca i Alimentació, 10.13039/501100002809Generalitat de Catalunya.

## Declaration of competing interest

The authors report there are no competing interests to declare.

## Data Availability

Data will be made available on request.
